# Implications of oral dysbiosis and HPV infection in head and neck cancer: from molecular and cellular mechanisms to early diagnosis and therapy

**DOI:** 10.3389/fonc.2023.1273516

**Published:** 2023-12-18

**Authors:** Marian Constantin, Mariana Carmen Chifiriuc, Grigore Mihaescu, Corneliu Ovidiu Vrancianu, Elena-Georgiana Dobre, Roxana-Elena Cristian, Coralia Bleotu, Serban Vifor Bertesteanu, Raluca Grigore, Bogdan Serban, Catalin Cirstoiu

**Affiliations:** ^1^ Department of Microbiology, Institute of Biology of Romanian Academy, Bucharest, Romania; ^2^ The Research Institute of the University of Bucharest, ICUB, Bucharest, Romania; ^3^ Microbiology Immunology Department, Faculty of Biology, University of Bucharest, Bucharest, Romania; ^4^ Department of Life, Medical and Agricultural Sciences, Biological Sciences Section, Romanian Academy, Bucharest, Romania; ^5^ DANUBIUS Department, National Institute of Research and Development for Biological Sciences, Bucharest, Romania; ^6^ Immunology Department, “Victor Babes” National Institute of Pathology, Bucharest, Romania; ^7^ Department of Biochemistry and Molecular Biology, Faculty of Biology, University of Bucharest, Bucharest, Romania; ^8^ Cellular and Molecular Pathology Department, Ştefan S. Nicolau Institute of Virology, Bucharest, Romania; ^9^ Coltea Clinical Hospital, ENT, Head & Neck Surgery Department, Carol Davila University of Medicine and Pharmacy, Bucharest, Romania; ^10^ University Emergency Hospital, Carol Davila University of Medicine and Pharmacy, Bucharest, Romania

**Keywords:** HNC, risk factors, signaling pathways, oral microbiota, HPV infection, tumor microenvironment

## Abstract

Head and neck cancer (HNC) is the sixth most common type of cancer, with more than half a million new cases annually. This review focuses on the role of oral dysbiosis and HPV infection in HNCs, presenting the involved taxons, molecular effectors and pathways, as well as the HPV-associated particularities of genetic and epigenetic changes and of the tumor microenvironment occurred in different stages of tumor development. Oral dysbiosis is associated with the evolution of HNCs, through multiple mechanisms such as inflammation, genotoxins release, modulation of the innate and acquired immune response, carcinogens and anticarcinogens production, generation of oxidative stress, induction of mutations. Thus, novel microbiome-derived biomarkers and interventions could significantly contribute to achieving the desideratum of personalized management of oncologic patients, regarding both early diagnosis and treatment. The results reported by different studies are not always congruent regarding the variations in the abundance of different taxons in HNCs. However, there is a consistent reporting of a higher abundance of Gram-negative species such as *Fusobacterium, Leptotrichia, Treponema, Porphyromonas gingivalis, Prevotella, Bacteroidetes, Haemophilus, Veillonella, Pseudomonas, Enterobacterales*, which are probably responsible of chronic inflammation and modulation of tumor microenvironment. *Candida albicans* is the dominant fungi found in oral carcinoma being also associated with shorter survival rate. Specific microbial signatures (e.g., *F. nucleatum, Bacteroidetes* and *Peptostreptococcus*) have been associated with later stages and larger tumor, suggesting their potential to be used as biomarkers for tumor stratification and prognosis. On the other hand, increased abundance of *Corynebacterium, Kingella, Abiotrophia* is associated with a reduced risk of HNC. Microbiome could also provide biomarkers for differentiating between oropharyngeal and hypopharyngeal cancers as well as between HPV-positive and HPV-negative tumors. Ongoing clinical trials aim to validate non-invasive tests for microbiome-derived biomarkers detection in oral and throat cancers, especially within high-risk populations. Oro-pharyngeal dysbiosis could also impact the HNCs therapy and associated side-effects of radiotherapy, chemotherapy, and immunotherapy. HPV-positive tumors harbor fewer mutations, as well as different DNA methylation pattern and tumor microenvironment. Therefore, elucidation of the molecular mechanisms by which oral microbiota and HPV infection influence the HNC initiation and progression, screening for HPV infection and vaccination against HPV, adopting a good oral hygiene, and preventing oral dysbiosis are important tools for advancing in the battle with this public health global challenge.

## 1 Introduction

Head and neck cancers (HNCs) encompass a group of malignancies that predominantly originate in the squamous cells (HNSCC) lining the upper aerodigestive mucosa (1) and rank as the sixth most common cancer globally, with an annual incidence of over half a million new cases, projected to exceed 900,000 new cases in 2020 (2).

**Figure 1:**
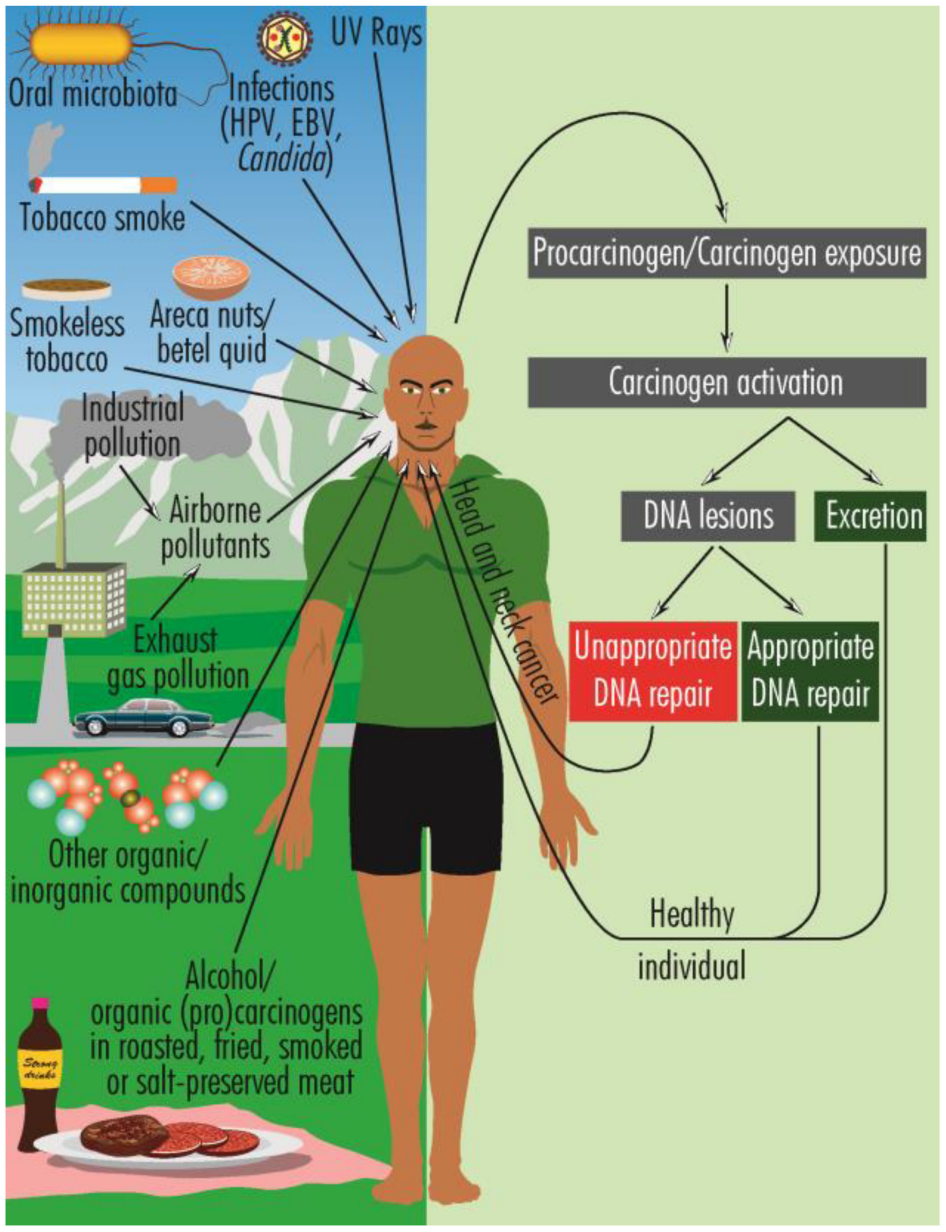
Schematic overview of the predisposing factors for HNCs and the general pattern of carcinogenesis. The activated carcinogens can produce DNA damage or are excreted. When the cellular repairing mechanisms are functioning properly, the DNA damage is repaired, but when these mechanisms are ineffective, genetic defects are perpetuated and can ultimately lead to HNCs.

HNCs have a multifactorial etiology, including genetic and epigenetic mechanisms (3), oral dysbiosis (4), infections with human papilloma virus (HPV), mostly oncogenic types 16 and 18 (5), and EBV (Epstein-Barr virus) (6), laryngopharyngeal reflux (7), prior exposure to radiotherapy (8) as well as various lifestyle features, such as heavy smoking and alcohol consumption (9, 10), chewing betel quid (Areca nuts) (11), marijuana use (12), poor oral hygiene (13, 14), pro-inflammatory diet (e.g., fried, smoked, or roasted meat) (15), oral dysbiosis (16), prolonged exposure to sunlight, inhalation of chemical pollutants (17–22) (**Figure 1**). These multifaceted risk factors highlight the need for comprehensive strategies in both prevention and management to combat HNCs effectively.

Infectious agents, including bacteria, fungi, parasites and viruses are known to cause an important percentage of the total number of cancers. Among viruses, HPV is one of the main risk factors for HNCs, the HPV infection being among the several biomarkers that can be used for the early diagnosis of HNCs (the viral DNA being found in approximately 4.5% of all cancers and 25% of HNCs (23–27).

Recent evidence shows that human microbiome and dysbiosis are also associated with HNCs and could provide novel biomarkers for getting one step closer to the desideratum of personalized management of oncologic patients. The oral dysbiosis has been associated with chronic inflammation (28), genotoxins release (29), generation of carcinogens or inhibition of anticarcinogenic compounds synthesis (30), favoring the occurrence of a pro-tumor local microenvironment and causing tumor growth (29). All these effects could be involved in the genesis or progression of different malignancies such as upper aerodigestive tract, esophagus, stomach, pancreas, colorectum, liver, lung and breast cancer (31).

Several papers recently described the role of HPV infection and the composition of the host microbiome in HNCs carcinogenesis (4, 28, 32–35). However, there is still a lack of understanding of the association between human microbiota signatures and the risk of HNCs, designating new biomarkers in HNCs diagnosis, and the implication of dysbiosis in HNCs therapy. Therefore, in this review, we discussed first the most recent original paper and clinical trials aiming to investigate the human microbiota signatures associated with HNC and human microbiota signatures associated with a reduced risk of HNC. Secondly, we discussed the potential microbiome-derived biomarkers for HNCs diagnosis and implications of oro-pharyngeal dysbiosis in HNCs therapy, presenting the results from the very recent studies over the past two years (36–53). Finally, we discussed the contribution of HPV infection to HNCs initiation and progress and implications for prevention, early diagnosis, and treatment.

## 2 Oral microbiota, dysbiosis and pathogenesis of HNCs

The oral cavity represents one of the most complex microbiomes in the human body (54), providing support and resources for an impressive number of microbial species (from 700-750 to several thousands) (28, 29), which include bacteria, archaea, fungi and protozoa (55), from at least 12 phyla (28), such as *Actinobacteria*, *Bacteroidetes*, *Firmicutes*, *Proteobacteria*, *Spirochaetes*, *Synergistetes* and *Tenericutes* (28). Oral microbes colonize both soft tissues (tongue, soft palate, oral mucosa, and tonsils) and hard tissues (teeth), developing specific biofilms (55). Under normal conditions, called eubiosis, the healthy microbiota shows relatively constant proportions of different taxons and stable diversity, which, in dysbiosis state, are disrupted in favor of commensal proinflammatory and pathogenic species (29).

### 2.1 Human microbiota signatures associated with HNCs

At phylum level, *Firmicutes*, *Proteobacteria*, *Bacteroidetes*, *Fusobacteria*, and *Actinobacteria* were the five most abundant phyla and accounted for > 90% of the bacterial community in aerodigestive tract cancers, including HNC (51). Other studies reveal a decrease of the phyla *Actinobacteria* and *Cyanobacteria* in HNCs (4, 29, 56).

At the genus level, *Streptococcus*, *Abiotrophia*, *Prevotella* and *Leuconostoc* were significantly reduced in the aerodigestive tract cancers group, *Haemophilus* increased, *while Neisseria* has been reported to have either high or low abundance (51, 57).

Other genera commonly associated with HNCs are *Fusobacterium*, *Leptotrichia*, *Selenomonas*, *Treponema*, *Clostridium*, and *Pseudoalteromonas* (40). In oral squamous cell carcinoma (OSCC), an increased abundance of *Parvimonas*, *Fusobacterium* (including *F. nucleatum*, which is reported to be the most abundant species in OSCCs samples, and *F. periodonticum*), *Pseudomonas* (*Pseudomonas aeruginosa* is reported to be the second most abundant species in OSCCs), *Porphyromonas gingivalis* (reported in gingival squamous cell carcinoma), *Peptostreptococcus*, *Alloprevotella*, *Capnocytophaga*, *Prevotella*, *Bacteroidetes* and *Solobacterium*, *Actinomyces*, *Lactobacillus, Rothia*, *Haemophilus* and *Veillonella* are reported (4, 29, 56–59).

Certain aerobic and facultative anaerobic bacteria, including *Klebsiella*, *Citrobacter*, *Streptococcus*, *Enterobacter*, and *Serratia*, have been found to affect the local tumor microenvironment in oral carcinoma (4).

*Candida albicans* is the dominant fungi found in oral carcinomas, a strong association between higher *Candida* carriage and a notably shorter overall survival (OS) being observed in patients with OSCC (60).

The abundance of different microbial species may change during tumor progression, and possible microbiota signatures can be associated with tumor stages and prognosis. High levels of serum class G antibodies against *F. nucleatum* were found in patients with gastrointestinal cancer and HNCs. However, in HNSCC developed by non-smokers, the abundance of *F. nucleatum* is associated with early tumor stages, but with reduced likelihood of recurrence, and increased survival duration (36).

During the progression of OSCC from stage I to IV, the abundance of *F. periodonticum*, *Parvimonas micra*, *Streptococcus constellatus*, *Haemophilus influenzae*, and *Filifactor alocis* gradually increases, while *Actinobacteria* phyla and *Streptococcus mitis*, *Haemophilus parainfluenzae* and *Porphyromonas pasteri* decrease (56, 61). Decrease of *Parvimonas* and increase of *Fusobacterium* (especially that of *F. nucleatum*), *Rothia*, *Haemophilus*, *Veillonella*, and *Actinomyces* is associated with early stages of tumor development (36, 56, 62, 63). A comprehensive systematic review concluded that *F. nucleatum* is present and in higher abundance in oral cancer samples when compared to non-cancer samples, suggesting that could contribute to oral cancer development (64). However, it is also possible that tumor colonization by *F. nucleatum* reflects its ability to exploit and replicate effectively in the hypoxic tumor microenvironment. To date, several *F. nucleatum*-carcinoma mechanisms have been discovered: promotion of the Wnt/β-catenin signaling pathway through FadA binding to E-cadherin (65), inhibition of the cytotoxicity of immune cells such as NK cells and T-cell activity (66), LPS binding to TLR4/MYD88 pathway and mediating downstream NF-κB expression (67) and the release of Fap2 that binding to Gal-GalNAc ligands (68). This evidence that *F. nucleatum* colonization begins early in the process of malignant transformation supports a potential role for microbiome changes in the pathogenesis of the disease (64). Consequently, Coppenhagen-Glazer and collaborators found that *F. nucleatum* is a key organism between early and late colonisers and its outer membrane adhesin Fap2 is partly responsible for facilitating multispecies biofilm formation (69). Also, it was revealed that the enrichment of *F. nucleatum* in OSSC is associated with host gene promoter methylation, including hypermethylation of tumor suppressor genes *LXN* and *SMARCA2*, a gene involved in ATP-dependent chromatin remodeling related to DNA repair and replication. This suggests that *F. nucleatum* enrichment may cause cell proliferation through epigenetic silencing (70).

Increasing abundance of *Bacteroidetes* and *Peptostreptococcus* has been associated with later stages and larger tumors (61). In terms of subsequent development, the presence of higher numbers of *Stenophotromonas*, *Staphylococcus*, *Centipeda*, *Selenomonas*, *Alloscordovia*, and *Acinetobacter* genera in the saliva of individuals with OSCC is associated with poor prognosis and poorer survival rate (63). Dou and collaborators observed that increased numbers of *Schlegelella* and *Methyloversatilis* in HNCs are associated with poor prognosis, while abundant *Bacillus*, *Lactobacillus*, and *Sphingomonas* are found in patients with favorable prognosis (37, 71).

Salivary bacteria, such as *S. salivarius*, *Corynebacterium*, and *Stomatococcus*, are associated with a strong oxidative stress that may contribute to oncogenesis and cancer development (4). The presence of *P. gingivalis* in the oral cavity tend to be associated with higher mortality rates (61, 72, 73). *P. gingivalis* is known to stimulate the production of myeloid-derived dendritic suppressor cells, which can inhibit the activity of cytotoxic T lymphocytes, a key component of the antitumoral immunity. Additionally, this bacterium can induce the overexpression of matrix metalloproteinase-9 and reduce the expression of the tumor suppressor gene TP53, thereby promoting cell proliferation and potentially contributing to cancer development (74). Some members of oral microbiota are metabolizing alcohol to acetaldehyde, a potent carcinogen and are reducing the synthesis of anticarcinogenic compounds, including siderophore group non-ribosomal peptides, 12-, 14- and 16-membered macrolides and monoterpenoids (30), The abundance of microorganisms in the phyla *Actinobacteria* is associated with mutations in *TP53*, while high numbers of *Firmicutes* with recurrent mutations in *FAT1*, *FZR1*, *AXIN1* and *WNT* (29).

*C. albicans* was also discovered to play a role in accelerating the progression of OSCC *in vitro*. This acceleration was attributed to several mechanisms, including the increased synthesis of matrix metalloproteinases and oncometabolites, the promotion of pro-tumor signaling pathways, and the upregulation of genes associated with prognostic markers for metastatic events (44, 75). Given these insights, there is potential for interventions targeting *C. albicans* to serve as a therapeutic strategy for HNC, offering promising avenues for developing novel treatments.

### 2.2 Human microbiota signatures associated with a reduced risk of HNC

The abundance of microorganisms of the genera *Corynebacterium*, *Kingella* (especially *K. denitrificans*), *Neisseria*, *Abiotrophia*, and *Capnocytophaga* is associated with a reduced risk of laryngeal cancers and the increased abundance of *Actinomyces (A. oris)*, *N. sicca* and *Veillonella denticariosi* species with a reduced risk of pharyngeal and other HNCs (29, 74). Many commensal bacteria from the genera *Corynebacterium* and *Kingella* appear to have a preventive effect on developing HNCs (76), and the abundance of *Veillonella* is associated with better overall prognosis of OSCCs (63). In 2018, a nested case-control study including 129 HNC patients, revealed that *Corynebacterium* and *Kingella* can reduce the risk of developing HNCs by contributing to the breaking down and neutralization of harmful toxic substances, including compounds like toluene, styrene, and chlorobenzene (76).

Another recent nested case-control study aiming to investigate the relationship of oral microbiome with HNC demonstrated that the presence of oral fungi and relative abundance of multiple microbial species, including the red- and orange-complex periodontal pathogens (*C. albicans*, *K. oralis*, *P. gingivalis*), were associated with reduced risk of HNC (53).

The relationships between several types of HNCs and microorganisms is summarized in **Table 1**.

**Table 1:** Association of some types of HNCs with microorganisms present in the oral cavity and their outcomes.

Cancer type	Taxon	Associated impact	Outcome	Reference
HNCs	*Fusobacterium*, *Leptotrichia*, *Selenomonas*, *Treponema*, *Clostridium*, *Pseudoalteromonas*	Common	?	(40)
	*Corynebacterium*, *Kingella*	Presence	Preventive effect	(76)
	oral fungi	Presence	reduced risk	(53)
	red- and orange-complex periodontal pathogens	Presence and relative abundance	reduced risk	(53)
HNSCC, non-smokers	*Fusobacterium nucleatum*	Presence	Early tumor stages Reduced recurrence Increased survival duration	(36)
OSCC	*Parvimonas*	Increased	?	(56)
	*Fusobacterium, Pseudomonas*		?	(58)
	*Peptostreptococcus*, *Alloprevotella*, *Capnocytophaga*, *Prevotella*, *Bacteroidetes*, *Solobacterium*,		?	(29) (56)
	*Actinobacteria*, *Cyanobacteria*, *Streptococcus*, *Porphyromonas*, *Actinomyces*, *Rothia*, *Haemophilus* and *Veillonella*	Decreased	?	
	*Fusobacterium periodonticum*, *Parvimonas micra*, *Streptococcus constellatus*, *Haemophilus influenzae*, *Filifactor alocis*	Gradually increased during stages I–IV	?	(12, 24, 53, 56, 77–82-61)
	*Actinobacteria* phyla, *Streptococcus mitis*, *Haemophilus parainfluenzae*, *Porphyromonas pasteri*	Gradually decreased during stages I–IV	?	
	*Parvimonas*	Decreased	Early tumor stages	(56) (36) (62) (63)
	*Fusobacterium*, *Rothia*, *Haemophilus*, *Veillonella*, *Actinomyces*	Increased		
	*Bacteroidetes*, *Peptostreptococcus*	Increased	Later stages Larger tumors	(61)
	*Stenophotromonas*, *Staphylococcus*, *Centipeda*, *Selenomonas*, *Alloscordovia*, *Acinetobacter*	Increased in saliva	Poor prognosis Poorer survival rate	(63)
	*Veillonella*		Better overall prognosis	
Laryngeal cancer	*Corynebacterium*, *Kingella* (especially *Kingella denitrificans*), *Neisseria*, *Abiotrophia*, *Capnocytophaga*	Abundance	Reduced risk	(29)
Pharyngeal cancer	*Actinomyces oris*, *Veillonella denticariosi*	Increased	Reduced risk	(29)
Gingival squamous cell carcinoma	*Porphyromonas gingivalis*	Increased	?	(29)
Oral mucosal cancer	*Streptococcus constellatus*, *Streptococcus mitis*, *Streptococcus oralis*, *Streptococcus sanguis*, *Streptococcus salivarius*	Translocated to lymph nodes	Drainage of microorganisms to lymph nodes and global circulation	(83) (31)
HNC, aerodigestive tract cancer	*Leuconostoc*, *Streptococcus*, *Abiotrophia*	Increased	Increased risk of all cancers	(51)
	*Prevotella*, *Haemophilus*, *Neisseria*	Decreased	Lowest risk of all cancers	
Oral carcinoma	*Klebsiella*, *Citrobacter*, *Streptococcus*, *Enterobacter*, *Serratia*	Increased	Affecting the local tumor microenvironment	(4)
	*S. salivarius*, *Corynebacterium*, *Stomatococcus*	Presence	Strong oxidizing properties; Oncogenesis and cancer development	
HNCs	*Schlegelella* and *Methyloversatilis*	Presence and relative abundence	Poor prognosis	(37, 71)
	*Bacillus*, *Lactobacillus*, *Sphingomonas*		Favorable prognosis	
OSCC	*C. albicans*	Presence	Accelerate progression; promotion of pro-tumor signaling pathways; upregulation of genes associated with prognostic markers for metastatic events	(44, 75)
Oral carcinoma	*P. gingivalis*	Presence	Stimulate the production of myeloid-derived dendritic suppressor cells; Reduce the expression of the tumor suppressor gene *TP53*; cancer development	(74)
oropharyngeal and hypopharyngeal cancers	*S. anginosus*	Significant elevation	non-invasive diagnostic biomarker	(84)
oropharyngeal cancers	*Haemophilus* and *Gemella*	Significant elevation	distinct microbiome profiles in cancer group	(80)
oral cancer	*Streptococcus*, *Haemophilus*, and *Actinomyces*	Downregulated	Potential-microbiome biomarkers in HNC diagnosis	(85)
oral cancer	*Fusobacterium*, *Prevotella*, *Alloprevotella*	Significant elevation	oral microbiota was extensively changed	(86)
oropharyngeal cancers	*Faecalibacterium*, *Prevotella*, *Phascolarctobacterium*	Significant elevation	lower risk of tumor recurrence	(32)
oral and throat cancers	*Actinobacillus*, *Mannheimia*, *Streptobacillus*	Presence	increased severity of oral mucositis (OM)	(87)
oral cancer	*Fusobacterium, Haemophilus*	Significant elevation	increased susceptibility to inflammatory complications	(88)
HNCs	*Eubacterium*, *Victivallis*, and *Ruminococcus*	Presence	increased severity of OM	(89)
	*Faecalibacterium*, *Prevotella*, and *Phascolarctobacterium*	Presence	better treatment outcomes	

### 2.3 Potential microbiome-derived biomarkers for HNCs diagnosis

In HNC, the current standard of screening and diagnosis relies on a physical exam and identification of lesions, followed by imaging, invasive biopsy, and histopathological evaluation (90). Current research aims to investigate and establish new microbiome signatures as potential microbiome-derived biomarkers for HNC diagnosis (45, 61, 84, 85, 91).

Differences in the microbiome profile between oropharyngeal and hypopharyngeal cancers were observed, with *S. anginosus* showing significant elevation in the saliva of oropharyngeal cancer patients (84). Mutational changes influence the abundance of bacterial groups like *Firmicutes* and *Bacteroidetes*, varying among different mutation profiles (61). In HPV-positive oropharyngeal cancers, a pilot study revealed distinct microbiome profiles compared to healthy controls, with a notable correlation between *Haemophilus* and *Gemella* genera in HPV-positive oropharyngeal cancer (80). Furthermore, certain bacterial species, including *Actinomyces*, *Parvimonas*, *Selenomonas*, and *Prevotella*, were more abundant in oral cavity cancers (91). At the species level, *S. salivarius* and *S. vestibularis* were identified as abundant in oral OSCC samples, while species from the vaginal microbiota, such as *L. gasseri / johnsonii* and *L. vaginalis*, were abundant in saliva (92). Banavar and collaborators conducted a study including 242 patients with oral cancer, aiming to develop and investigate machine-learning classifiers using metatranscriptomic data from saliva samples. The developed metatranscriptomic signatures incorporated both taxonomic and functional microbiome features, and revealed several taxa and functional pathways associated with oral cancers. The authors observed that several genera, such as *Streptococcus*, *Haemophilus*, and *Actinomyces*, are downregulated, while some other genera, like *Fusobacterium*, do not appear to be differentially expressed. At the genus level, the results revealed periodontal bacteria like *Fusobacterium*, *Prevotella*, and *Porphyromonas* in saliva samples from oral cancers. More recently, the same research group conducted a clinical trial aiming to develop and validate a non-invasive test for biomarkers detection in oral and throat cancers within a high-risk population. The authors collected saliva samples from 1175 patients and used machine learning methods to obtain a salivary microbial and human metatranscriptomic signature. This developed test, named CancerDetect for Oral and Throat Cancer (CDOT), has received the FDA’s breakthrough designation for accelerated review (45). These studies demonstrated the potential of a machine-learning tool for oral cancer diagnosing, opening a new era of non-invasive diagnostics, enabling early intervention, and improving patient outcomes.

In addition, Inchingolo and collaborators conducted a meta-analysis to evaluate the interplay between microbiota and oral cancer and the presence of biomarkers as risk predictors. The analysis of the results from 21 studies revealed the correlation between oral cancers and changes in the microbiota, which explains the paramount value of precision medicine in the diagnosis and treatment of HNCs (47). Ganly and collaborators observed that oral microbiota was extensively changed in oral cancer patients due to the increase of periodontal pathogens like *Fusobacterium*, *Prevotella*, and *Alloprevotella* and the reduction in commensal *Streptococcus*. Based on these marker genera, the oral microbiota was split into two types: periodontal-pathogen-low and periodontal-pathogen-high. This classification predicted oral cancer with 80% accuracy. In addition to the three periodontal pathogens discovered in the samples, the cumulative abundance of 14 periodontal pathogens increased gradually throughout the sequence of negative controls. These data consistently indicate that periodontal infections are an independent risk factor in patients who do not have substantial oral risk factors (86). Furthermore, in 5 patients with OSCC, saliva metaproteomics indicated a substantial rise in *Prevotella* and the adhesion and virulence factors linked to *S. gordonii*, as well as oral pathogens like *Fusobacterium* (93). In a study by Li and collaborators, the microbial composition in three distinct groups of samples from patients with oral cancer was investigated using metagenomic sequencing. The study found that while there was limited variation in the microbial diversity of the three groups, the oral microbiome of patients with precancerous lesions exhibited greater diversity than that of both oral cancer patients and healthy controls. Notably, a specific strain of *Bacteroidetes* within the phylum displayed differential enrichment in the oral cancer samples. Furthermore, at the genus level, the primary differentially enriched taxa included *Prevotella*, *Peptostreptococcus*, *Carnobacterium*, and *Diastella*. *P. intermedia* and *Peptostreptococcus stomatis* were identified as having distinct species-level enrichment patterns, suggesting that these profiles can be employed as diagnostic markers (67). A rise in potentially pathogenic bacteria, such as *Capnocytophaga*, and other LPS-producing bacteria, such as *Neisseria*, were seen in the oral microbiome of 56 HNC patients. The study concluded that HNC-related symptoms in conjunction with salivary microorganisms such as *Capnocytophaga* may be employed as a noninvasive technique for screening, identification, and treatment monitoring of HNC (94). In patients with OSCC, significant increase of *Fusobacterium* and a concomitant reduction in *Firmicutes* and *Actinobacteria* phyla have been found. Significant distinctions were also revealed in *Actinobacteria*, *Firmicutes*, *Fusobacteriia*, *Fusobacteriales*, *Fusobacteriaceae*, and *Fusobacterium*. These findings brought into light five unique oral microorganisms with high confidence and may be used to predict clinical diagnosis and prognosis (48).

### 2.4 Implications of oro-pharyngeal dysbiosis in HNCs therapy

The composition of a patient’s gut microbiota impacts the effectiveness and side effects of radiotherapy, chemotherapy, and immunotherapy, playing a significant role in HNC outcomes. A prospective pilot study including 20 HNC patients has shown that a pre-treatment microbiota enriched with *Eubacterium*, *Victivallis*, and *Ruminococcus* is associated with a higher risk of experiencing OM, a common side effect of cancer treatment that affects the mouth and throat. Conversely, when the gut microbiota has a higher relative abundance of immunomodulatory microbes such as *Faecalibacterium*, *Prevotella*, and *Phascolarctobacterium*, patients are at a lower risk of tumor recurrence (32). These microbes seem to play a role in modulating the response to immunotherapy by potentially enhancing the expansion and function of CD8+ T cells, which are crucial for mounting an effective antitumor immune response. However, it is essential to note that more extensive research is required to validate these associations and determine whether modifying the gut microbiota can predict and optimize treatment outcomes for HNC patients. Dysbiosis has been shown to promote the persistence of ulcers and delay the healing process (88). The presence of certain bacteria, such as *Actinobacillus*, *Mannheimia*, and *Streptobacillus*, has been associated with increased severity of OM (87). *Fusobacterium* and *Haemophilus*, when dominant in the oral microbiome before radiotherapy, are associated with an increased susceptibility to inflammatory complications. Specific bacteria, including *Prevotella*, *Fusobacterium*, *Streptococcus*, *Megasphaera*, and *Cardiobacterium*, have been considered as prognostic biomarkers for the onset of OM (88). Research by Jiang and collaborators demonstrated that patients who received probiotics during chemoradiotherapy experienced a lower incidence of oral mucositis compared to those who did not receive probiotics (95). Similarly, a study by Ma and collaborators found that patients who received probiotic therapy were more likely to complete radiotherapy without complications, in contrast to those without probiotics, where patients had to discontinue treatment due to complications (96). The research conducted by Al-Qadami and collaborators has revealed significant associations between specific bacterial genera and the severity of OM and treatment outcomes in cancer patients. Three bacterial genera, namely *Eubacterium*, *Victivallis*, and *Ruminococcus*, were found to be linked to more severe OM. On the other hand, the presence of bacterial genera *Faecalibacterium*, *Prevotella*, and *Phascolarctobacterium* was associated with better treatment outcomes (89). In conclusion, modifying the gut microbiota to align with more favorable treatment outcomes represents a promising avenue for future research and clinical practice.

According to Routy et al., patients who do not respond well to immunotherapy often have gut dysbiosis (97). Other studies show that addition of dietary supplementation with *Bifidobacterium* appears to have a comparable effect on tumor control compared to treatment with a specific antibody therapy targeting programmed death-ligand 1 (PD-L1). Furthermore, when combined, these therapies nearly eliminated the expansion of tumors, suggesting a synergistic or enhanced therapeutic effect (98). Similarly, *Lactobacillus* and *Bacteroides* species could trigger type I interferon production in dendritic cells, enhancing the cross-priming of antitumor CD8+ T cells having different impacts on the immunostimulatory effects (43). Additionally, those who have undergone antibiotic therapy, particularly immediately before or during cancer treatment, face a higher risk of rapid disease progression (97).

The severity of oral injuries in patients undergoing radiation therapy, such as changes in saliva quantity and composition, alterations in the oral microbiota, and tooth damage, is primarily linked to the radiation dose delivered to the oral cavity region (99). Radiation-induced acidification of the oral environment creates a favorable condition for the proliferation of acidogenic and cariogenic bacteria, such as *S. mutans*, *Actinomyces*, and *Lactobacillus*, while reducing the populations of *Neisseria*, *Fusobacterium*, and *S. sanguinis*. Untreated, these caries-associated bacteria contribute to developing radiation therapy-related dental caries (87). Furthermore, *C. albicans* can take advantage of these shifts in the oral microbiota, potentially leading to superinfections during and after therapy (99). A study by Huo et al. prospectively evaluated the dynamic changes in oral microbiota during radiation therapy and its association with the progression or aggravation of oropharyngeal mucositis in a cohort of nasopharyngeal carcinoma patients. The results showed that while the overall richness and evenness of mucosal bacterial diversity did not vary significantly during treatment, certain bacteria, such as *Prevotella*, *Fusobacterium*, *Treponema*, and *Porphyromonas*, exhibited noticeable synchronized shifts in their abundance throughout radiation therapy. These shifts often coincided with the onset of severe mucositis, suggesting that dysbiosis of the oral mucosal microbiota may play a role in exacerbating mucositis in nasopharyngeal carcinoma patients during radiation therapy (99). A clinical study by Mougeot and collaborators investigated the oral microbiome implications in developing post-radiotherapy caries in 31 HNC patients. The results suggested that baseline microbiome difference is an essential factor explaining dental caries outcomes in radiation-treated HNC patients. Also, the cariogenic role of *P. melaninogenica* and a potential protective role of specific bacterial species such as *A. defectiva* was reported (100).

In HNC patients undergoing chemoradiotherapy, treatment is often associated with challenging side effects, such as mucositis and dysphagia, to which oropharyngeal microbiota might contribute, but the precise causal relationship and clinical significance have remained unclear. To shed light on this matter, a prospective longitudinal observational study involving 47 HNC patients was conducted to determine if dysbiosis is present in HNC and to assess the impact of chemoradiotherapy on the dynamics of dysbiosis during and after treatment. The salivary microbiome in the HNC patients before initiating treatment exhibited notable differences in composition and decreased diversity compared to a control group of healthy individuals. During treatment, there was a significant decrease in α-diversity and a marked shift in β-diversity, suggesting a significant change in microbial composition compared to the pre-treatment state and healthy controls. The microbiome analysis showed no significant difference in α-diversity between HNC patients with severe mucositis and those with mild to moderate mucositis before treatment. However, marked differences in α-diversity emerged immediately after the completion of chemoradiotherapy (52). Omega-3 (ω-3) polyunsaturated fatty acids have recently gained a particular interest in dealing with oral diseases owing to their anti-inflammatory, antioxidant, and wound-healing properties (101). In a recent clinical study conducted by Morsy and collaborators, 34 HNC patients received radiotherapy and topical Omega-3 nanoemulgel. A significant reduction in *Firmicutes/Bacteroidetes* ratio was observed after six weeks in the test group, indicating less microbial dysbiosis. The results demonstrated that topical omega-3 nanoemulgel has a beneficial effect in preventing radiation-induced OM with the possibility of regulating oral microbial dysbiosis (50).

The success of immune checkpoint inhibitors in the context of palliative systemic therapy for HNSCC and the potential for combining these immunotherapies with radiotherapy have brought to the forefront the exploration of interactions between the tumor microenvironment and the immune landscape (39, 102). It has become increasingly clear that the regulation of the immune system by the microbiota is of paramount importance for both innate and adaptive immune surveillance against tumors and for the success of treatment with these immune checkpoint inhibitors (39, 103).

### 2.5 Microbiome-based interventions for managing cancer therapy-related side effects

Recently, a meta-analysis conducted by Frey-Furtado and colleagues examined nine articles to evaluate the therapeutic effectiveness of probiotics in managing OM. Among these studies, four clinical trials reported a decrease in the severity of OM by using specific strains of bacteria, including *Lactobacillus* (*L. casei* and *L. brevis CD2*) and *B. clausii UBBC07*. Preclinical studies revealed the positive effects of *L. lactis*, *L. reuteri*, and *S. salivarius K12* in reducing the severity of OM and the size of ulcers (46). Moreover, three distinct meta-analyses conducted by research teams from Taiwan, Italia and China, encompassing a total of 22 randomized clinical trials, investigated the potential of probiotics in preventing OM induced by cancer therapy and in managing the occurrence of chemotherapy-induced diarrhea and OM. These studies revealed the effectiveness of probiotics in preventing and alleviating cancer therapy-induced OM and addressing adverse reactions associated with chemotherapy (38, 41, 49).

*Lactobacillus rhamnosus GG* (LGG) is a naturally occurring gut commensal bacterium known for its anti-inflammatory properties and has been a pioneer in oncology research (104). LGG maintains the equilibrium of the intestinal mucosa by neutralizing harmful pathogens and toxins, effectively preventing breaches in the mucosal barrier through a high-affinity binding system (105). LGG is also recognized for enhancing the anticancer effects of geniposide, an anticancer molecule, and its potential as a beneficial adjuvant during cancer treatment (106). In the context of cancer treatment, *Lactobacillus brevis CD2* lozenges have been found to reduce the occurrence of OM in patients undergoing high-dose chemotherapy (107). Additionally, *L. brevis* lozenges have shown benefits in reducing oral ulcers in individuals with recurrent aphthous stomatitis (108).

Xerostomia, a condition characterized by dry mouth, has a detrimental impact on the oral health of many patients undergoing radiotherapy for HNSCCs. In a pilot study, Vesty and collaborators explored the potential of using an oral probiotic to influence the oral bacterial community following radiotherapy positively. The authors conducted a four-week intervention involving oral probiotic lozenges containing *Streptococcus salivarius M18* in seven patients and compared the changes in oral health and the composition of bacterial communities in plaque and saliva with a control group of six patients who received a placebo. Both groups improved periodontal screening and plaque index scores after the intervention. Surprisingly, the oral probiotic did not lead to significant alterations in the composition or diversity of bacterial communities in the oral cavity. Network analyses revealed potential negative interactions between administered probiotics and bacteria from genera known for their association with periodontal disease, such as *Campylobacter*, *Fretibacterium*, *Selenomonas*, and *Treponema* (109).

In a comprehensive meta-analysis, Lu and the research team investigated the impact of oral probiotics on the management of side effects induced by radiotherapy, chemotherapy, or chemoradiotherapy in cancer patients. Their study analyzed data from 16 randomized controlled trials involving 2,097 patients. The study’s findings revealed that when compared to placebo groups, the use of oral probiotics (*Bifidobacterium longum,B. infantis, Lactobacillus acidophilus*, *Bacillus clausii*, *L. plantarum*, *L. rhamnosus*, *L. crispatus*, *Enterococcus faecium*) yielded significant reductions in the occurrence of side effects associated with radiotherapy and chemotherapy across various cancer types, including HNSCCs. Additionally, the analysis indicated that the incidence of OM in HNSCCs patients significantly decreased following the oral administration of probiotics (42).

In a randomized clinical trial conducted by Doppalapudi and their research team, the primary objective was to evaluate the impact of probiotic bacteria on oral *Candida* counts in cancer patients undergoing head and neck radiotherapy at a tertiary care center. The study involved randomly allocating participants into three equal-sized groups: the probiotics group, the candid group, and the combination group. Participants in the probiotics group were administered probiotic sachets containing a minimum of 1.25 billion live cells consisting of a blend of four probiotic strains, namely *L. acidophilus*, *L. rhamnosus*, *Bifidobacterium longum*, and *Saccharomyces boulardii*. The study results unveiled a statistically significant reduction in the mean counts of *Candida* species (measured in colony-forming units per milliliter, CFU/ml) after the intervention. This notable reduction was primarily observed in both the probiotics group and the combination therapy group. Furthermore, besides a decrease in *C. albicans*, there was a significant reduction in *C. glabrata* and *C. tropicalis* following probiotic usage compared to the other groups (110). These findings strongly suggest that probiotic bacteria effectively reduce the presence of oral *Candida* species and could be recommended as a standalone approach or combined with traditional antifungal agents to effectively reduce oral *Candida* in patients undergoing head and neck radiotherapy.

## 3 HPV viral components and molecular genetic and epigenetic mechanisms involved in HNCs

From the >220 HPV viruses at least 12 are oncogenic (111, 112). Of all HNC cases caused by chronic, persistent HPV infection, approximately 85% are positive for the HPV16 or HPV18 types. The remaining approximately 15% are caused by HPV33, HPV35, HPV52, HPV45, HPV39, HPV58, HPV53, and HPV56 (26, 27). The HPV6, HPV11, HPV16, HPV18, HPV31, HPV33, HPV45, HPV52, and HPV58 strains are accounting for 90% of HNC cases (24, 26, 113). The percentage of HPV positivity varies with the type of HNCs and the different geographic regions (26, 27, 113–117), the highest incidence being reported for sub-Saharan African region (HPV has been identified in 50% of oropharyngeal cancers, 27% of laryngeal cancers, and 23% of oral cavity cancers, with the predominance of HPV16 (118).

### 3.1 Genetic peculiarities of HPV-positive and HPV-negative HNCs

**Figure 2:**
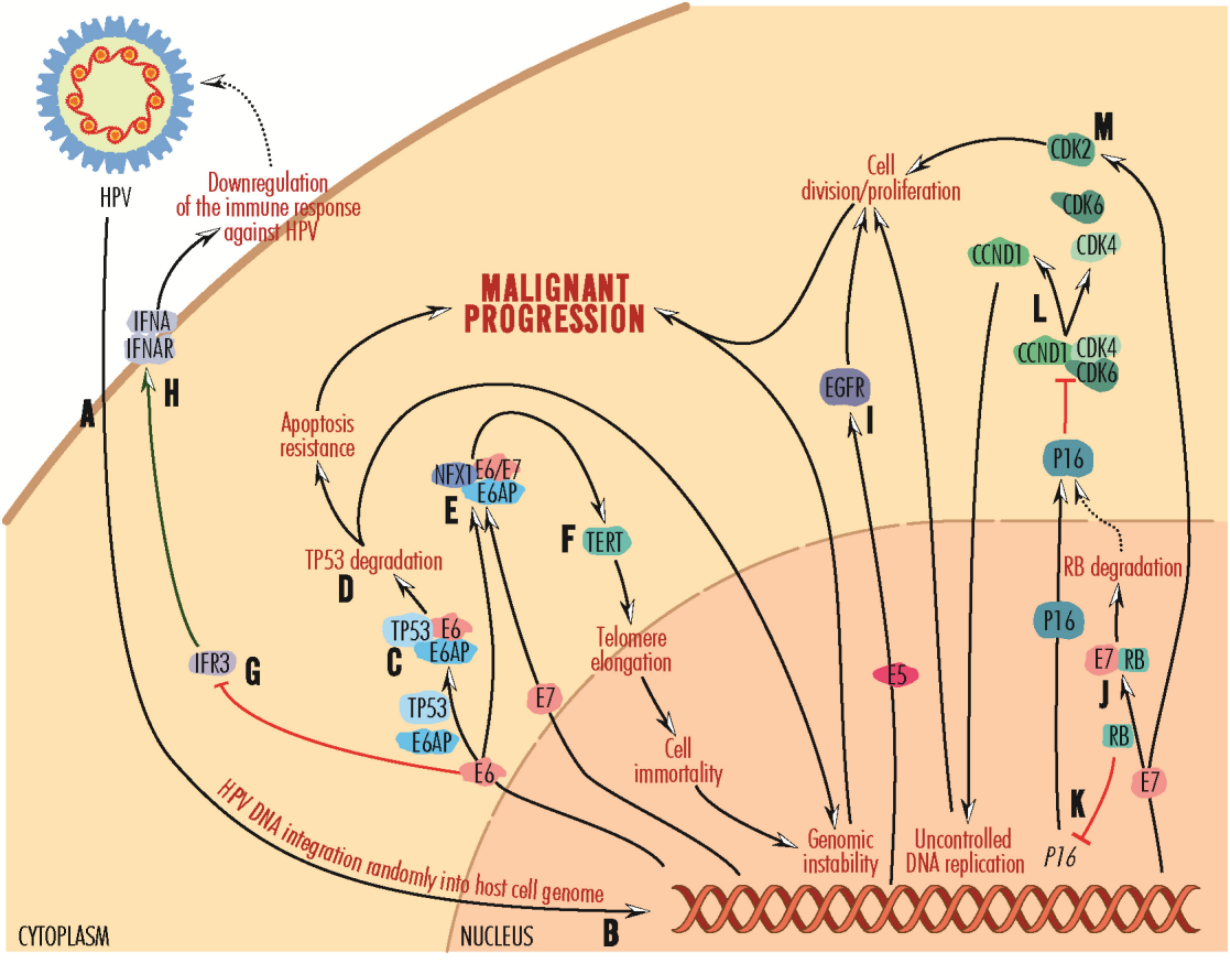
Mechanisms of carcinogenesis induced by persistent HPV infections. Viral particles infect epithelial cells in the oral or oropharyngeal mucosa **(A)**, with HPV DNA randomly integrating into the host cell genome **(B)**. It is replicated as the epithelial cells multiply, and the virus is activated when it reaches the surface. After integration into the host cell genome, viral DNA is copied into mRNA, and proteins are released into the nucleus and cytoplasm. The E6 protein recruits the cellular ubiquitin-protein ligase E6AP and targets the cellular protein TP53 **(C)**, which is involved in maintaining the genetic health of cells. Complexed with E6 and E6AP, TP53 protein is degraded in proteasomes **(D)**, an event that promotes resistance to apoptosis and malignant progression. Lacking the DNA integrity checkpoint mechanism, the cell can accumulate defects, leading to genomic instability and malignant progression. On the other hand, the E6 protein, and less E7, forms a complex with E6AP and NEX1 (neurogenic differentiation factor 6) **(E)**, which activates TERT/hTERT **(F)**. This telomerase reverse transcriptase promotes telomere elongation and cell immortalization. Further, cells with inactive TP53 due to proteasomal degradation may acquire genetic instability and be transformed toward malignant progression. The E6 protein inactivates IFR3 **(G)**, normally promoting the IFNA-IFNAR complex **(H)** formation. By inhibiting the formation of this complex, E6 decreases immune recognition of HPV and helps the spreading of HPV infection. The E5 protein activates the EGFR-mediated signaling pathway **(I)**, promoting cell division and proliferation toward malignant progression. The E7 protein forms a complex with RB, leading to proteasome degradation **(J)**. In the absence of RB, P16 synthesis is activated **(K)**, which binds and disrupts CCND1 complexes with CDK4 and CDK6 **(L)**, CCND1 contributing to uncontrolled DNA replication and cell division, which can further lead to malignant progression. The E7 protein stimulates CDK2 activity **(M)**, leading to cell proliferation and malignant progression.

Of the HPV viral components, the nonstructural E (early) proteins E5, E6 and E7 are associated with virus-mediated cellular transformation, the most active and expressed in HPV-positive tumor cells being E6 and E7 (27, 119). These viral proteins once accumulated intracellularly can initiate the carcinogenic process, affect the immune system, alter the activity of tumor suppressor proteins (e.g., E6 binds the TP53 protein *via* the cellular ubiquitin-protein ligase E6AP/E3A or UBE3A, mediating its proteasomes degradation) and circumvent cell-cycle checkpoints (5, 120) (**Figure 2**). TP53 activity is modulated by MDM2 (mouse double minute 2, also named retained in humans). According to an *in silico* study by Bouzid and collaborators, in HPV-positive HNSCC, MDM2 is overexpressed compared to HPV-negative tumors (33). The *TP53* gene is only rarely mutated in HPV-positive, but very frequently in HPV-negative tumors, in which disruptive mutations are associated with reduced survival (27, 121). Actually, the number of mutations in HPV-positive tumors is twice as low as in HPV-negative tumors (122).

HPV-positive tumors have TpC transversions and structural alterations of RNA and DNA, including insertion of the viral genes *E6*, *E7*, and *E2F1* (the latter being amplified) and, in some cases, defects in the *TRAF3* (TNF Receptor Associated Factor 3) gene. Defects in *TP53* and *CDKN2A* genes are absent or rare, with frequent alterations in *PIK3CA*, *PTEN* (phosphatase and tensin homolog), *FBXW7* (F-Box and WD Repeat Domain Containing 7), and *KRAS* genes.

HPV-positive tumors include two subtypes: HPV–KRT, with amplification of the 3q region, presence of mutations in *PIK3CA*, and overexpression of genes involved in keratinocyte differentiation (*CDH3*–Cadherin 3, and *TP63*– Transformation-Related Protein 63/Tumor Protein 63) and oxidation-reduction processes (*CDH1* and *KRT16*), and HPV-IMU, characterized by deletion of the 16q region, differentiation of mesenchymal cells dictated by the BCL2 gene, activating mutations of the *PIK3CA* gene and a strong immune response based on activation of the *NFKB* (Nuclear Factor Kappa B), *RELB* (RELB Proto-Oncogene, NF-KB Subunit) and *FOXP3* (Forkhead Box P3) genes (123).

Amplification of the 7p region containing the *EGFR* (Epidermal Growth Factor Receptor) gene, encoding a transmembrane receptor in the RAS–RAF–MEK–ERK and PIK3–AKT–mTOR signaling pathways, is also absent (124–128).

In HPV-positive premalignant tissue, the SYCP2 (synaptonemal complex protein 2), involved in the organization of chromatin is up-regulated (5), and in HPV-positive HNSCC recurrent mutations have been identified in the tumor suppressor genes PTEN and TRAF3, and in the PIK3CA (phosphatidylinositol-4,5-bisphosphate 3-kinase catalytic subunit alpha) gene, which promotes carcinogenesis (129). Also, Hinic and collaborators identified overexpression of PCNA (proliferating cell nuclear antigen) genes, associated with cell proliferation and transformation in cancer, *TNFRSF14* (TNF receptor superfamily member 14), which promotes inflammatory and inhibitory T cell immune response, *TRAF1* (TNF receptor-associated factor 1), *TRAF2* (TNF receptor-associated factor 2), which mediate anti-apoptotic and pro-survival signals from TNF receptors, *BIRC3* (baculoviral IAP repeat containing 3) and *BCL2* (B-cell lymphoma 2), with anti-apoptotic functions (130). A set of genes associated with the extracellular matrix-receptor interaction pathway, which include *ITGA5* (integrin alpha five subunits), *ITGB1* (integrin beta 1 subunit), *LAMB1* (laminin beta 1 subunit), and *LAMC1* (laminin gamma 1 subunit), are overexpressed in HPV-positive HNCs (and cervical cancers). Genes associated with T lymphocyte function, *CD3D* (CD3 delta subunit of T-cell receptor complex), *CD3E* (CD3 epsilon subunit of T-cell receptor complex), *CD8B* (CD8 beta subunit), *LCK* (LCK proto-oncogene, SRC family tyrosine kinase), and *ZAP70* (zeta chain of T-cell receptor-associated protein kinase 70kDa), are underexpressed in HPV-positive HNCs and cervical cancers. The observed dysregulation in the latter set of genes in both cancers indicates some expression specificity related to HPV infection, showing a significant prognostic impact on HPV-associated cancers (131).

The HPV-negative tumors frequently harbor CpG transversions, defects in *TP53*, *CCND1*, *MYC*, miR let-7c, *TP63*, and *AJUBA* genes, amplification of *EGFR*, *ERBB2* (Erb-B2 Receptor Tyrosine Kinase 2), *FGFR1* tyrosine kinase receptor genes, deletions in *NSD1* (Nuclear Receptor Binding SET Domain Protein 1), *CDKN2A*, *NOTCH1*, *SMAD4* (SMAD Family Member 4), *FAT1* genes, *NFE2L2*, *KEAP1* (Kelch Like ECH Associated Protein 1), *CUL3* (Cullin 3), *KMT2D/MLL2*, *HLA-A/CMH-IA*, and co-amplifications of 11q13, with *CCND1*, *CTTN* (Cortactin) and *FADD* (Fas Associated *via* Death Domain) genes, and 11q22, with YAP1, *BIRC2* (Baculoviral IAP Repeat Containing 2), and *CASP8*+/–*HRAS* genes, in some subsets of HPV-negative tumors (128). In a small number of HNCs, the *MET* (MET Proto-Oncogene, Receptor Tyrosine Kinase) gene transcript with missing exon 14, a form recognized as oncogenic in non-small cell lung cancer, and mRNAs of *TP63* and *KLK12* (Kallikrein Related Peptidase 12) genes as specific splicing variants have been reported (126). The harbor frequent inhibitory mutations in TP53 and the CDKN2A/B deletion associated or not with CCND1 amplification occur are leading to G1/S checkpoint abrogation (129). Activating mutations in the *NOTCH1* gene and increased transcription of the *FGF1* (Fibroblast Growth Factor 1) gene result in increased cell migration and invasiveness and increased mortality in patients with oral cancers (77). Depending on the presence or absence of gene copy number amplification, HPV-negative tumors are divided into two main subtypes: tumors without copy number amplification or class “M” (from mutations) tumors, which occur in the oral cavity and in which mutations are reported in the HRAS and CASP8 genes as well as in mismatch repair genes (in tumors developed by those who chew betel quid), but lack the TP53 gene, and tumors with copy number amplification. These are divided into three subtypes, basal, classical, and mesenchymal, found in frequent smokers. The basal subtype is characterized by deletions in the 9p arm, which includes the *CDKN2A* gene (9p21. 3), amplifications of genes in the 3q arm, 11q13/q22 co-amplification, coexistence of mutations in the *HRAS* and *CASP8* (Caspase 8) genes, inactivation of *NOTCH1*, which has an oncogenic function but in HNCs appears to have a tumor suppressor function, and reduced activity of the *SOX2* (SRY-Box Transcription Factor 2) gene (3q26.33). The classical subtype is present in high proportion in laryngeal squamous cell cancers, shares with the basal subtype the presence of deletions in the 9p arm, with loss of *CDKN2A* and apposition of genes in the 3q arm, and is characterized by mutations in the *TP53* gene, changes in *KEAP1*, *CUL3* and *NFE2L2* genes, involved in oxidative stress management. In the mesenchymal subtype, epithelial-mesenchymal transitions, amplification of the 3q region, activation of the WNT-β-catenin pathway, mutations of the *HLA-A/CMH-IA* gene, and increased activity of the *CD56/NCAM1* (Cluster of differentiation 56/Neural Cell Adhesion Molecule 1), *VIM* (Vimentin), *DES* (Desmin), *TWIST1* (Twist Family BHLH Transcription Factor 1) and *HGF* (Hepatocyte Growth Factor) genes predominate. In both HNCs, amplification of the 3q26/28 region has been identified with *TP63*, *SOX2*, and *PIK3CA* genes, the product of the latter being part of the PIK3–AKT–mTOR signaling pathway with an important role in cell proliferation and evasion of apoptosis (128).

E6 also contributes to the downregulation of the immune response against HPV by suppressing IFR3 (Interferon Regulatory Factor 3), a transcription factor for interferons (IFNs) (132, 133), and by inhibiting IFNA (interferon alpha) interaction with its receptor (134). E7 acts synergically by inhibiting TLR9/CD289 (Toll-like receptor-9), present intracellularly in several immune cell types (135, 136).

Also, by stimulating CDK2 (cyclin-dependent kinase 2) activity and inactivating its inhibitors, P21CIP1 and P27KIP1, E7 supports cell division (135) and tumorigenesis. Viral protein E7 binds and degrades RB (retinoblastoma) tumor suppressor cell proteins (137). Further, RB inactivation induces expression of P16/P16INK4 (cyclin-dependent kinase inhibitor protein 16), which binds and disrupts the CCND (cyclin D) and CDK4 (cyclin-dependent kinase 4)/CDK6 (cyclin-dependent kinase 6) complexes, amplifying cyclin D1 and promoting uncontrolled DNA replication and the transition of cells from G1 to S phase (138).

In addition, E6 and E7 (to a lesser extent) are involved in stimulating cell division and cell immortalization by promoting TERT/hTERT (telomerase reverse transcriptase) expression, which is repressed in normal cells and elongates telomeres through replication and by preventing end-to-end-fusion-of-chromosomes-and-cell apoptosis.

P16 expression and HPV status influence the prognosis of oropharyngeal tumors. HPV+ and P16+ oropharyngeal tumors have a better prognosis than HPV+ and P16– or HPV– and P16+ tumors, while HPV– and P16– tumors have the poorest prognosis (139).

E5 mediates hyperactivation of the EGFR-mediated signaling pathway, stimulating cell proliferation (140).

Another mechanism by which viral proteins inhibit cell apoptosis is by blocking the FAS/FASL (Fas cell surface death receptor/Fas cell surface death receptor ligand) pathway and binding to TNFR1 (tumor necrosis factor receptor 1).

Depending on the affinity of viral proteins for inhibited cellular proteins, there are low-risk HPV strains and high-risk HPV strains that develop tumors. For example, the low-risk HPV11 is generally suppressed by innate immunity, and when this is overcome, it can cause benign lesions (5). On the contrary, high-risk strains, such as HPV16 and HPV18, are not suppressed by the innate immune system and are free to develop long-term infections and produce the majority of HNCs (27). However, HPV infections alone do not appear to be sufficient for the tumorigenic transformation of cells, this process requiring new mutations induced by other risk factors, including smoking and alcohol consumption, or infection with other oncoviruses, such as the polyomaviruses BKV and JCV (John Cunningham virus), the simian vacuolating virus 40 (SV40) and the most likely Epstein-Barr virus type B (141–145).

This could explain the different clinical manifestations of oropharyngeal cancer in HPV-positive versus HPV-negative patients (146, 147). Primary HPV-positive tumors are small in size but develop frequent, more extensive lymph node metastases (148), with frequent immune infiltrates rich in CD8+ cytotoxic T lymphocytes and PDL1 overexpression compared to HPV-negative tumors (82). Correlated with P16 expression, CD8+ T lymphocyte accumulation in the tumor microenvironment improves overall survival in OSCC (82, 149).

### 3.2 Contribution of HPV infection to the epigenetic changes occurring in HNCs

In addition to gene expression changes induced by nucleotide sequence alterations, in neoplastic development, an important role is also played by gene expression changes induced by epigenetic alterations, including DNA methylation, posttranslational covalent histone modifications, and non-coding RNA (150, 151). Methylation occurs through the DNMTs (DNA methyltransferases) activity in the presence of AdoMet (S-adenosylmethionine) as a cofactor. In cancer, DNMTs activities are altered, with tumor suppressor genes being silenced by hypermethylation while numerous other sequences spread throughout the genome are hypomethylated, leading to DNA double helix fragmentation and genomic instability (152). In HNCs, the methylation range of different genes is variable. It has been reported that there are significant differences in terms of DNA methylation between HPV-positive and HPV-negative HNCs, due to the fact that E6 and E7 viral proteins interfere with cellular DNA methylation complexes. For example, the inactivation of TP53 by E6 protein stops repression of DNMT1 (DNA (cytosine-5)-methyltransferase 1) promoter, altering the global cytosine methylation pattern (153, 154). Also, changes in cellular DNA-methylation machinery lead to altered gene expression. The affected gene classes are genes involved in cell cycle regulation and programmed cell death: *CDKN2A* (cyclin-dependent kinase inhibitor 2A, tumor suppressor which is hypomethylated in salivary samples of HPV-positive HNC patients) (155, 156), *RASSF1* (Ras association domain family 1, tumor suppressor which is hypomethylated in NHCs) (156, 157), *CCNA1* (cyclin A1, whose promoter is hypermethylated in HNCs) (155, 156); genes involved in cellular adhesion and communication: Cadherin Family Genes (involved in cell adhesion and playing important roles in cell signaling and communication, whose pşromoters are hypermethylated) (124, 156, 158), *ITGA4* (integrin alpha 4, hypermethylated) (159, 160); genes involved in cellular migration and tumor progression: *TIMP3* (tissue inhibitor of metalloproteinase and tumor suppressor, whose promoter methylation is reported in a few studies in HPV-driven HNCs) (78, 161), *ELMO1* (engulfment and cell motility 1 protein, which is relatedto increased invasion and metastasis in several types of cancer and hypermethylated in HNCs) (81, 160); other genes: *MEI1* (meiotic double-stranded break formation protein 1), whose promoter is hypomethylated in HNCs) (81, 124), and *LINE1* (long interspersed nuclear element 1, an abundant retrotransposome found in human genome, which is hypermethylated in HNCs) (155, 162, 163). In HPV-negative tumors, LINE1 is hypomethylated (162). Also, the HPV integrated genomes become subjects of DNA methylation/hypermethylation (164). Even some studies have reported similar findings in matter of HPV-driven DNA-methylation signatures, making proposals for using them as biomarkers for HNCs, these patterns have not been comprehensively investigated. Their inconsistence requires further investigations for identifying the diagnostic methylation targets for HNCs (164).

Little progress has been made in using DNA demethylation as an approach in HVP-positive HNCs. Thus, Stich and collaborators reported that the demethylating agent 5-aza-2’-deoxycytidine can reduce *E6* and *E7* gene expression in HPV-infected HNCs and cervical cell lines effectively, with some restoration of TP53 and P21 function and increased tumor suppressor microRNA 375 levels, contributing to overall decrease in cancer cell growth and survival (165). These results lead to the conclusion that the ability to reverse DNA methylation makes it an attractive target for drug intervention in HNCs (independent of HPV status), unlike mutations and deletions, which are quite more difficult to correct (166, 167).

### 3.3 Influence of HPV infection on tumor microenvironment features in HNCs tumorigenesis

The tumor microenvironment is a complex entity that comprises diverse cellular components and extracellular matrix constituents and, through bidirectional interaction with tumor cells, can contribute to tumor progression (168). The cellular component of the tumor microenvironment is very diverse and comprises genetically transformed stromal cells, including endothelial cells, adipocytes, cancer-associated fibroblasts, peripheral nervous system-derived nerve fibers, blood or lymphatic cells, infiltrating immune cells (T lymphocytes, B lymphocytes, and NK cells), neuroendocrine cells, macrophages, neutrophils, antigen-presenting dendritic cells, and myeloid-derived suppressor cells. The immune component of the tumor microenvironment comprises cytotoxic T lymphocytes, regulatory T lymphocytes, B lymphocytes, NK cells, macrophages, neutrophils, antigen-presenting dendritic cells, and myeloid-derived suppressor cells infiltrating the tumor stroma. The NK cells target and induce apoptosis in transformed cells and virus-infected cells that escape the action of cytotoxic T lymphocytes. Macrophages maintain tumor progression by secretion of activating cytokines, angiogenesis, and metastasis. Myeloid-derived suppressor cells promote tumor growth, inhibition of T lymphocyte cytotoxicity, tumor angiogenesis, disintegration of extracellular matrix by secretion of MMPs, inhibition of NK cell activity, and activation of regulatory T lymphocytes with immunosuppressive function (169, 170). The tumor-associated B lymphocytes can trigger humoral antitumoral immunity through interactions with regulatory T lymphocytes and dendritic cells. Dendritic cells are among the most potent antigen-presenting cells, providing cytotoxic CD8+ T lymphocytes with recognition keys to tumor targets (171, 172).

In HNSCC, CD56dim NK cell infiltration is markedly higher in HPV-positive tumors and probably contributes to their more favorable prognosis. NK cells recognize unhealthy or foreign cells that expose inappropriate HLA/MHC class I molecules. Activation of NK cells requires that the proportion of activating signals exceeds that of inhibitory signals and occurs directly *via* membrane receptors in two pathways and indirectly *via* soluble factors. The first direct activation pathway requires binding FCGRIIIA/CD16, one of the most potent NK cell activating receptors, to the Fc region of immunoglobulins. The low affinity of the FCGRIIIA/CD16 receptor allows NK cells to recognize and release immunoregulatory cytokines against antibody-coated cellular targets (173). The second pathway of direct NK cell activation occurs *via* NKG2D and NCRs (Natural Cytotoxicity Receptors), such as NKP30 and NKP46, and is strongly induced under stress conditions in viral infections, including HPV infection and in tumor cells (174, 175). Indirect activation occurs *via* soluble cytokines, including the interleukins IL2, IL12, IL15, IL18, and IL21, TNFA (Tumor Necrosis Factor Alpha), and IFN (type I interferon) (170, 176).

Neutrophils are among the first immune cells recruited in infections (177) and inflammation in the microenvironment of HNSCC (178). Infiltration of neutrophils in the tumor stroma and an elevated neutrophil to lymphocyte ratio is associated with poor overall surviving for HNSCC patients (179). In HNSCC, the neutrophil increase is lower in HPV-positive than in HPV-negative tumors. However, in the former, an increased neutrophil count is associated with reduced survival duration (180).

Dendritic cells infiltrating HNSCC of the tonsil are of two types: plasmacytoid CD123+ dendritic cells, with characteristics of lymphocytes and classical dendritic cells, myeloid CD11c+ dendritic cells, with three subtypes, CD1c+ myeloid dendritic cells, CD141+ myeloid dendritic cells, and CD1c-CD141- myeloid dendritic cells (181). HNSCC significantly reduces the number of CD11c+ myeloid dendritic cells in the peripheral circulation, which increases after tumor resection. Due to antigen-presenting activity to T lymphocytes, CD1a+ myeloid dendritic cell clusters in the stroma of HNSCC are associated with favorable prognosis and increased survival duration, with some studies indicating them as a favorable prognostic marker for some HPV-positive but not HPV-negative tumors, but this is not always the case (182).

Cytotoxic CD8+T lymphocytes are the main cellular immune effectors against tumor cells. Activation of cytotoxic CD8+ T lymphocytes occurs through TCR (T cell receptor) recognition of HLA/MHC antigens presented by dendritic cells and interaction of co-stimulatory factors B7/CD80 on antigen-presenting cells and CD28 on T lymphocytes. CD28 activates CTLA4/CD152, expressed predominantly on cytotoxic CD8+ T lymphocytes and less on activated B lymphocytes, monocytes, dendritic cells, regulatory CD4+ T lymphocytes, and granulocytes, induces TGFB (Transforming Growth Factor Beta) synthesis with immunosuppressive effects (183). In tumor tissue, TGFB synthesis leads to overexpression of CTLA4/CD152, with depletion of T lymphocytes (184), which begin to release inhibitory molecules, including PD1, CTLA4, TIGIT (T Cell Immunoreceptor With Ig And ITIM Domains) and LAG3 (Lymphocyte Activating 3), which reduce their activity and production of cytokines and cytolytic molecules (185). PD1 is part of the CD28 family of receptors and has PDL1 and PDL2 ligands, and both are expressed on antigen-presenting cells, endothelial cells, and activated lymphocytes (186).

Increased expression of PD1 and PDL1 in the tumor microenvironment cells leads to the inactivation or depletion of cytotoxic CD8+ T lymphocytes, and even when these cells are present in large extent, it favors tumor survival (187). HPV infections are known to increase the number of specific CD 8+ T lymphocytes, whose proliferation is triggered mainly by L1 protein (188). In a similar manner, HPV-positive tumors attract an increased number of HPV-specific CD 8+ T lymphocytes, which account 0.1 to 10% among all the tumor-infiltrating CD8+ T lymphocytes, while their presence in peripheral blood is very low (0.02%), indicating a strong association with the tumor microenvironment. One subset of HPV-specific CD 8+ T lymphocytes is expressing the *TCF7* and other genes associated with with PD1+ stem-like CD8 T lymphocytes, which are very important for maintaining T cell responses when HPV-antigen presence is prolonged. When stimulated with the HPV peptides, the PD1+TCF1+ stem-like subset of CD8+ T lymphocytes proliferates and differentiates into several subsets of effector cells, and the presence of functional and proliferative HPV-specific PD1+TCF1+CD45RO+ stem-like CD8 T proves that in HPV-positive NHC tumors there is active mechanisms to overcome the PD1 blockade (189), leading to chronic inflammatory reaction and poor prognosis in OSCC wif’th high expression of PD1 and its ligands (190, 191). On the other hand, 82 show that PDL1 expression on macrophages infiltrating HPV-positive tumors indicates a trend toward improved overall survival. Since HPV-positive HNCs are able to develop responsive mechanisms to PD1 blockade, the PD1–PDL1 pair could be an attractive target for antitumor therapies (192). In some cancer cases, PD1/PDL1 antibody therapies invigorate tumor-infiltrating CD8+ T lymphocytes, but their efficacy on heterogeneous CD8+ T cell populations is uneven (193). However, the tumor response to PD1 inhibitor therapy depends on tumor type. It is expected that, in the presence of CD8+ T lymphocytes, PD1+ tumors are more responsive compared to PD1-negative tumors (194–196), and anti-tumor therapeutic decisions may be guided by the results of immunohistochemical tests for PDL1 expression (197).

## 4 Discussion

HNCs continue to be a global health challenge and a topic of significant contemporary importance. HNCs are aggressive tumors, with more than 90% of their origin in squamous cells from the mucosae of the upper aerodigestive tract, ranking sixth among the most common cancers. Recent advances in omics and bioinformatics technologies are vital for gaining insights into the biology and clinical behavior of HNCs, unveiling potential biomarkers and therapeutic targets with practical applications in this problematic disease (198–201).

The dysbiosis occurred in the complex oral microbiome is associated with the evolution of HNCs, through multiple mechanisms such as inflammation, genotoxins release, modulation of the innate and acquired immune response, of carcinogens and anticarcinogens productions, generation of oxidative stress, induction of mutations (34). Thus, novel microbiome-derived biomarkers and interventions could significantly contribute to achieving the desideratum of personalized management of oncologic patients, regarding both early diagnosis and treatment.

**Figure 3:**
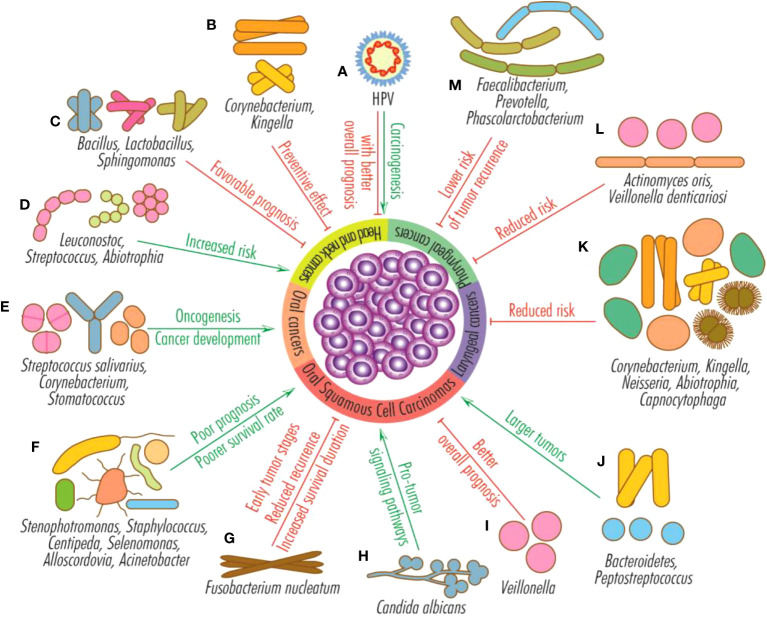
Effects of HPV infection and microbiota on head and neck tumors. HPV infection favors the development of head and neck cancers (mainly from the oral and oropharyngeal sphere), but their general prognosis is better than that of HPV-negative tumors **(A)**; *Corynebacterium* and *Kingella* have a preventive effect on head and neck cancers **(B)**; *Bacillus*, *Lactobacillus* and *Sphingomonas* sustain favorable prognosis **(C)**; *Leuconostoc*, *Streptococcus* and *Abiotrophia* increase the risk of head and neck cancers **(D)**; *Streptococcus salivarius*, *Corynebacterium* and *Stomatococcus* favor oncogenesis and the development of oral tumors **(E)**; *Stenophotromonas*, *Staphylococcus*, *Centipeda*, *Selenomonas*, *Alloscordovia* and *Acinetobacter* predict poor prognosis and poorer survival rate for OSCCs **(F)**; *Fusobacterium nucleatum* is generally associated with early oral squamous cell carcinomas stages, reduced recurrence and increased survival duration **(G)**; the fungus *Candida albicans* stimulates pro-tumor signaling pathways **(H)**; *Veillonella* is an indicator for better overall prognosis for OSCCs **(I)**; *Bacteroidetes* and *Peptostreptococcus* are associated with large oral squamous cell carcinomas **(J)**; the presence of the genera *Corynebacterium*, *Kingella*, *Neisseria*, *Abiotrophia* and *Capnocytophaga* reduce the risk of the appearance or progression of laryngeal tumors **(K)**; the species *Actinomyces* oris and *Veillonella denticariosi* are associated with reduced risk of occurrence or progression of pharyngeal tumors **(L)**; species from the genera *Faecalibacterium*, *Prevotella* and *Phascolarctobacterium* reduce the risk of pharyngeal cancer recurrence **(M)**.

The most common microorganisms associated with HNCs are *Porphyromonas gingivalis, Fusobacterium, Leptotrichia, Selenomonas, Treponema, Parvimonas, Pseudoalteromonas, Prevotella, Alloprevotella, Capnocytophaga, Bacteroidetes, Solobacterium, Clostridium* and *Peptostreptococcus*. A higher abundance of Bacteroidetes and *Peptostreptococcus* are associated with later stages and larger tumors, while increased salivary levels of *Stenophotromonas, Staphylococcus, Centipeda, Selenomonas, Alloscordovia*, and *Acinetobacter* with poor prognosis and poorer survival in oral cancer (**Figure 3**).

However, the results reported by different studies are not always congruent regarding the variations in the abundance of different taxons in HNCs. Thus, *Actinobacteria* phylum and *Neisseria, Capnocytophaga, Veillonella* genera are reported either with high or with low abundance in HNCs. The current studies are consistent in reporting a higher abundance of Gram-negative species such as *Fusobacterium, Leptotrichia, Treponema, Porphyromonas gingivalis, Prevotella, Bacteroidetes, Haemophilus, Veillonella, Pseudomonas, Enterobacterales*, which are probably responsible of chronic inflammation and modulation of tumor microenvironment. On the other side, a recent study shows that the presence of oral fungi and of red- and orange-complex periodontal pathogens was associated with reduced risk of HNCs. *C. albicans* is the dominant fungi found in oral carcinoma being also associated with shorter survival rate. The abundance of different microbial species such as *F. nucleatum, Bacteroidetes* and *Peptostreptococcus* has been associated with later stages and larger tumor, suggesting their potential to be used as biomarkers for tumor stratification and prognosis.

On the other side, some microbiota signatures, such as the abundance of microorganisms of the genera *Corynebacterium, Kingella, Abiotrophia* are associated with a reduced risk of HNCs.

Microbiome could also provide biomarkers for HNCs diagnosis, the profiles being different between oropharyngeal and hypopharyngeal cancers as well as between HPV-positive and HPV-negative tumors. Ongoing clinical trials aim to validate non-invasive tests for microbiome derived biomarkers detection in oral and throat cancers, especially within high-risk populations. These studies demonstrate the potential of machine-learning tools for oral cancer diagnosing, opening a new era of non-invasive diagnostics, enabling early intervention, and improving patient outcomes.

Oro-pharyngeal dysbiosis could also impact the HNCs therapy and associated side-effects of radiotherapy, chemotherapy, and immunotherapy, such as OM or tumor recurrence.

Elucidation of the molecular mechanisms by which oral microbiome and HPV infection influences the HNCs initiation and progression, screening for HPV infection and vaccination against HPV, adopting good oral hygiene, and preventing oral dysbiosis are important tools for advancing in the battle with this public health global challenge. Reducing pathogenic bacteria and promoting a healthy microbiome has been shown to enhance the effectiveness of both immunotherapy and chemotherapy. Furthermore, maintaining a balanced gut microbiome can help mitigate the side effects associated with these treatments, improving the overall experience for cancer patients (202, 203).

Current studies investigate the potential of probiotics in modulating the course of the disease and managing cancer therapy-related side effects in HNSCC and OSCC patients (38, 41, 42, 46, 49, 204). The current evidence underscores the potential of probiotics such as LGG and *L. brevis* in alleviating cancer associated oral mucosal problems and promoting overall health.

However, the current findings raise questions about the beneficial properties of particular oral probiotics, necessitating further investigation to better understand their effects and potential drawbacks. While the results regarding the use of microbiome-based interventions are promising, further research is recommended, specifically advocating for additional randomized, double-blind, multicenter trials conducted on a more extensive and diverse population. This approach will help provide a more robust understanding of the potential benefits of probiotics in managing cancer therapy-related side effects, such as the inflammation of the oral mucosa frequently encountered in individuals undergoing radiotherapy and chemotherapy (205).

As we gain a better understanding of the molecular traits distinguishing HPV-positive from HPV-negative HNSCCs, there is hope for developing novel early diagnosis markers (e.g., with methylated circulating tumor DNA) and targeted, personalized therapies. The specific genetic and epigenetic events occurred in each HNCs stage are influenced by the HPV infection status. HPV-positive tumors are characterized by fewer mutations, occurring predominantly in *PIK3CA*, *PTEN*, *FBXW7*, and *KRAS* genes, whereas HPV-negative tumors carry defects in a more extensive number of genes. In addition to inhibitory and activating mutations, in HNCs, gene expression is altered by several epigenetic changes, which, in the case of DNA methylation, are also influenced by the HPV status. The HPV status also influences the tumor microenvironment cellular components, particularly the NK, neutrophils, dendritic cells, and CD8 positive T cells. However, despite notable advancements in comprehending the molecular mechanisms by which HPV influences the HNCs evolution and response to treatment, the significant disparity in survival and clinical outcomes between HPV-positive and HPV-negative HNSCC patients following standard-of-care treatment remains enigmatic. Several research teams have proposed potential factors, including the elevated rates of cell proliferation and DNA damage in HPV-positive tumors compared to their HPV-negative counterparts (206). Future studies that address aberrant DNA methylation, histone post-translational modifications, non-coding RNAs, dysbiosis, and approaches to minimize immunosuppression within the tumor microenvironment will provide the science-based evidence for revolutionizing HNCs managment (207). Personalized treatments, encompassing targeted therapies, immunotherapies, cancer vaccines, and epigenetic inhibitors tailored to each individual’s molecular profile, hold great promise in overcoming the limitations of conventional therapies, offering patients more effective and precisely tailored care in clinical settings.

## Author contributions

MC: Writing – original draft, Writing – review & editing. MCC: Conceptualization, Writing – original draft, Writing – review & editing, Funding acquisition. GM: Writing – review & editing. COV: Writing – original draft, Writing – review & editing. E-GD: Writing – review & editing. R-EC: Writing – review & editing. CB: Writing – review & editing. SB: Writing – review & editing. RG: Writing – review & editing. BS: Writing – review & editing. CC: Writing – review & editing.
